# Nicotinamide mononucleotide alleviates endotoxin-induced acute lung injury by modulating macrophage polarization via the SIRT1/NF-κB pathway

**DOI:** 10.1080/13880209.2023.2292256

**Published:** 2023-12-15

**Authors:** Simeng He, Xianhong Jiang, Jing Yang, Ya Wu, Jia Shi, Xiaoyang Wu, Shihan Du, Yuan Zhang, Lirong Gong, Shuan Dong, Jianbo Yu

**Affiliations:** a Department of Anesthesiology and Critical Care Medicine, Tianjin Nankai Hospital, Tianjin Medical University, Tianjin, China; b Tianjin Key Laboratory of Acute Abdomen Disease Associated Organ Injury and ITCWM Repair, Institute of Acute Abdominal Diseases of Integrated Traditional Chinese and Western Medicine, Tianjin Nankai Hospital, Tianjin, China

**Keywords:** NMN, septic lung injury, NAD^+^

## Abstract

**Context:**

Sepsis-induced acute lung injury (ALI) is a severe condition with limited effective therapeutics; nicotinamide mononucleotide (NMN) has been reported to exert anti-inflammatory activities.

**Objective:**

This study explores the potential mechanisms by which NMN ameliorates sepsis-induced ALI *in vivo* and *in vitro*.

**Materials and methods:**

Cultured MH-S cells and a murine model were used to evaluate the effect of NMN on sepsis-induced ALI. MH-S cells were stimulated with LPS (1 μg/mL) and NMN (500 μM) for 12 h grouping as control, LPS, and LPS + NMN. Cell viability, apoptotic status, and M1/2 macrophage-related markers were detected. The mice were pretreated intraperitoneally with NMN (500 mg/kg) and/or EX-527 (5 mg/kg) 1 h before LPS injection and randomized into 7 groups (*n* = 8): control, LPS, LPS + NMN, NMN, LPS + NMN + EX-527 (a SIRT1 inhibitor), LPS + EX-527, and EX-527. After 12 h, lung histopathology, W/D ratio, MPO activity, NAD^+^ and ATP levels, M1/2 macrophage-related markers, and expression of the SIRT1/NF-κB pathway were detected.

**Results:**

In MH-S cells, NMN significantly decreased the apoptotic rate from 12.25% to 5.74%. In septic mice, NMN improved the typical pathologic findings in lungs and reduced W/D ratio and MPO activity, but increased NAD^+^ and ATP levels. Additionally, NMN suppressed M1 but promoted M2 polarization, and upregulated the expression of SIRT1, with inhibition of NF-κB-p65 acetylation and phosphorylation. Furthermore, inhibition of SIRT1 reversed the effects of NMN-induced M2 macrophage polarization.

**Conclusions:**

NMN protects against sepsis-induced ALI by promoting M2 macrophage polarization *via* the SIRT1/NF-κB pathway, it might be an effective strategy for preventing or treating sepsis-induced ALI.

## Introduction

Sepsis-induced acute lung injury (ALI) is a leading cause of morbidity and mortality worldwide and imposes substantial economic, social, and health burdens (Grasselli et al. 2020). Despite advances in knowledge of septic pulmonary pathologies over the years, efficient targeted therapies are still lacking (Park et al. 2019; Xiong et al. 2020).

The progression of ALI is largely driven by inflammatory responses, in which macrophages are the predominant effector cells that play a critical role in determining the extent and severity of pulmonary inflammation (Vladimirovna et al. 2016; Chakraborty et al. 2017; Li et al. 2021). During acute lung injury, macrophages can be activated by various milieu, and the phenotypes exhibit high plasticity to differentiate into classically activated macrophages (M1) and alternatively activated macrophages (M2) (Vladimirovna et al. 2016). Promoting macrophage transformation towards the M2 phenotype may contribute to inflammatory resolution and tissue repair (Ardura et al. 2019). Therefore, inducing M2 polarization may represent a promising therapeutic intervention for sepsis-induced ALI.

Nicotinamide mononucleotide (NMN) is considered one of the precursors that best represents the biology and therapeutic potential of nicotinamide adenine dinucleotide (NAD^+^) (Zhao et al. 2021). Evidence indicates that NAD^+^ contributes to energy and metabolism homeostasis and mechanisms that affect cell survival in stress and toxicity (Ardura et al. 2019). The pharmacological activities in the spotlight of NMN involve cellular biochemical functions, cardio-protection, diabetes, and aging-related complications (Poddar et al. 2019). Recently, the roles of NMN in sepsis including reducing cellular inflammation, oxidative stress, and apoptosis has been recognized (Yoshino et al. 2018; Poddar et al. 2019). However, the protective mechanism of NMN remains incompletely understood, and little is known about the effect on macrophage polarization in acute lung injury.

In addition to cellular bioenergetics and metabolism, NAD^+^ plays a crucial role in catalysing deacetylation of sirtuins, which are sensitive to dynamic fluctuations in metabolism (García-Prat et al. 2017; Kane and Sinclair 2018). Sirtuins have been confirmed to be key regulators of sepsis through antioxidant, anti-apoptotic, and anti-inflammatory properties (Covarrubias et al. 2021). In this regard, Sirtuin 1 (SIRT1) was recently shown to deacetylate and inactivate transcription factors and coactivators to dampen the inflammatory cascade (Yang et al. 2021). The NF-κB pathway plays a central role in regulating the transcription of inflammatory cytokines and mediators during sepsis (Puri and Naura 2022). Herein, we speculated that the SIRT1/NF-κB axis is responsible for the protective effect of NMN against sepsis-induced ALI.

In the current study, a cultured mouse alveolar macrophage cell line (MH-S) and a murine model of ALI induced by intraperitoneal LPS administration were used to determine the efficacy of NMN and its potential underlying mechanisms. We investigated whether NMN exerts its function in endotoxin-induced ALI by inducing macrophage polarization to M2 phenotype *via* the SIRT1/NF-κB signalling pathway.

## Materials and methods

### Cell culture and treatment

MH-S cells were purchased from Solarbio Science and Technology Co., Ltd. (Beijing, China), an agent of Applied Biosystems. Cell lines were cultured in RPMI-1640 (Gibco, CA, USA) supplemented with 10% foetal bovine serum (Gibco, CA, USA) and 1% penicillin–streptomycin solution (Gibco, CA, USA) at 37 °C with 5% CO_2_.

Cells were maintained at approximately 80%–90% confluence and seeded at 10^5^ cells/mL density in cultured plates. To evaluate the role of NMN in MH-S cells, 1 μg/mL LPS (*E. coli*-L2630, Sigma, USA) and/or 125, 250, 500 μM NMN (M3501, Sigma, USA) were then added to the plates. After incubation for 12 h, adherent cells were collected for further analysis.

### Cell viability

Before being treated with different manipulations, MH-S cells were dissociated and resuspended at a density of 2 × 10^3^ cells/mL, seeded into 96-well plates (100 μL each well), and cultured overnight at 37 °C. Briefly, 10 μL of CCK-8 solution (Beyotime, China) was transferred to microplates and incubated for 2 h. Subsequently, cell viability was determined at OD450 nm with a microplate reader.

### Detection of apoptosis

The apoptosis of MH-S cells was assessed using an Annexin V-FITC/PI detection kit (Absin, Shanghai, China) following the instruction manual. MH-S cells were collected after dissociation and rinsed with prechilled PBS after 48 h in culture. Subsequently, the precipitate was collected in 300 μL binding buffer, followed by incubation with Annexin V-FITC (5 μL, 15 min) and later with PI (5 μL, 5 min) shielded from light. The intensity was measured using flow cytometry (EXFLOW 206, Dakewe, China) and analysed by FlowJo v10 software (BD Biosciences, USA).

### Animals and treatment

The experiment was approved by the Animal Ethics Committee in the Tianjin Nankai Hospital (Approval No. NKYY-DWLL-2019-012, Tianjin, China). Six-week-old (20 ∼ 25 g) male C57BL/6 mice were supplied by Experimental Animal Technical Co. Ltd. (Beijing, China; animal license number: SYXK (Jing)2017-0024) and maintained in a temperature-controlled facility (humidity: 40%–60%, temperature: 22–25 °C) with a 12-h light/dark cycle. The animals were randomly assigned into seven groups (*n* = 8): control, LPS, LPS + NMN, NMN, LPS + NMN + EX-527, LPS + EX-527, and EX-527. To establish the endotoxin-related ALI model, lipopolysaccharide (15 mg/kg, Sigma, USA) was injected intraperitoneally as reported previously (Shi et al. 2021). Mice were pretreated intraperitoneally with NMN (500 mg/kg, Sigma, USA) and/or SIRT1 inhibitor EX-527 (5 mg/kg, Beyotime, China) 1 h before LPS injection as previously described (Caton et al. 2011; Xu et al. 2016). Additionally, an equal volume of sterile saline served as a vehicle control. The mice were euthanized under deep anaesthesia *via* cervical dislocation after 12 h, and care was taken to reduce animal suffering. Serum was obtained by eyeball extirpation with centrifugation at 3000 rpm for 10 min for further analysis. Lung tissues were fixed in 4% paraformaldehyde or stored at −80 °C for further analyses.

### Lung wet-to-dry weight (W/D) ratio

The W/D ratio was measured to determine the degree of pulmonary oedema. After determining the wet weight (W), tissues were desiccated at 65 °C for 48 h to obtain the dry weight (D).

### Lung histological analysis

Histological evaluation of lung tissue was routinely fixed, dehydrated, embedded, and sliced into 4 μm thick sections, followed by H&E staining. The slices were visualized using a microscope (× 400 magnification, Leica, Germany). Semiquantitative evaluation (lung injury score) of ALI was based on inflammatory cell infiltration, interstitium widening, oedema, and haemorrhage (Li et al. 2021; Shi et al. 2021). Each characteristic was scored (0: normal to 4: very severe) by a pathologist blinded to the experimental setup.

### NAD^+^ and NAD^+^/NADH measurement

NAD^+^ concentrations from the lungs were measured with an NAD/NADH Assay Kit (KA1657, Abnova, China) according to the manufacturer’s protocols. For sample preparation, frozen lung tissue (20 mg) was homogenized and 100 μL NAD and NADH extraction buffer were added for NAD and NADH detection, respectively. The extracts were heated for 5 min at 60 °C, followed by 20 μL assay buffer and 100 μL negative buffer to centrifugated at 14000 rpm for 5 min, the supernatant was collected and used for detection of NAD and NADH levels. Subsequently, 61 μL assay buffer, 12 μL MTT, 1 μL NAD cycling enzyme mix, and 1 μL PES were mixed as working reagents, and 40 μL sample and 80 μL working reagent were added per well. The absorbance was measured at 565 nm, and the NAD^+^ concentration was calculated as the total NAD content minus the NADH content.

### ATP content

Pre-weighted lung tissue was processed for measurement of lung ATP content using a commercial kit (Beyotime, China). This ATP assay kit is based on the quantitative measurement of a stable level of light by luciferase-catalyzed enzymatic reactions. Approximately 20 mg of lung tissue was homogenized in 200 mL lysates and centrifuged at 12000 *g* for 5 min. The supernatant was added to 100 μL ATP assay buffer and then the ATP concentrations were calculated from the standard curve data and normalized to the corresponding tissue weight.

### Assessment of myeloperoxidase (MPO) activity

MPO activity from lung tissue was measured by an MPO detection kit (A044-1-1, Nanjing Jiancheng, China) following the instructions. Briefly, lung tissues were homogenized and the supernatant was incubated with the reaction buffer in a water bath at 60 °C for 10 min, then the OD 460 nm was evaluated by a spectrophotometer (Lambda 35, PerkinElmer, USA). The activities of MPO are presented as U/g of tissue.

### Flow cytometry analysis

MH-S cells and single-cell lung suspensions were prepared as previously described (Deng et al. 2020). Thereafter, all single cells were resuspended in staining buffer (BD Bioscience, San Diego, CA, USA) and then stained with the following monoclonal antibodies: PE-F4/80 antibody (cat. 565410, BD, USA), FITC-CD11b antibody (cat. 557396, BD, USA), APC-CD86 antibody (cat. 558703, BD, USA), and PerCP-CD206 antibody (cat. 141716, BD, USA). All single cells were incubated with antibodies in the dark for 15 min and washed with PBS. Data were collected using EXFLOW 206 (Dakewe, China) and analysed using FlowJo v10 software (BD, USA).

### Reverse-transcription polymerase chain reaction (RT-PCR)

Total RNA from the lungs was isolated by a Qiagen RNeasy kit (Hilden, Germany) and cDNA was performed with a cDNA synthesis kit (TaKaRa, China). The reverse transcription conditions were 37 °C for 15 min, 85 °C for 5 s, followed by 4 °C for 10 min (T100 Thermal Cycler, Bio-Rad, USA). The amplification conditions were 30 s at 95 °C for pre-denaturation, 5 s at 95 °C for 40 cycles of denaturation, 5 s at 95 °C for annealing and 34 s at 60 °C for extension (7500 real-time PCR system, Applied Biosystems, USA). Data were calculated through 2^−ΔΔCt^ method and the primers are listed in Table 1.

**Table 1.** Primer sequences for RT-PCR.

Genes	Forward sequence (5’-3’)	Reverse sequence (5’-3’)
GAPDH	CCTGGAGAAACCTGCCAAGTA	GGAAGAGTGGGAGTTGCTGTTG
TNF-α	GGCAGGTCTACTTTGGAGTCATTGC	ACATTCGAGGCTCCAGTGAATTCGG
IL-10	ACCTGGTAGAAGTGATGCCCCAGGCA	CTATGCAGTTGATGAAGATGTCAAA
IL-6	CCACTGCCTTCCCTACTTCA	TCTTGGTCCTTAGCCACTCC
IL-1β	GCTGCTTCCAAACCTTTGAC	AGCTTCTCCACAGCCACAAT
Arg1	AACACGGCAGTGGCTTTAACCT	GTGATGCCCCAGATGGTTTTC
iNOS	AGGAAGTGGGCCGAAGGAT	GAAACTATGGAGCACAGCCACAT

### Immunofluorescence

Lung slices were prepared as described above and incubated with anti-AceNF-κB-p65 (1:200, Thermo Fisher Scientific, PA5-114696, USA) primary antibody for 12 h at 4 °C and secondary antibody (1:200, Beyotime, China) for 1 h. Nuclear counterstaining was stained with 4′-6-diamidino-2-phenylindole (DAPI), followed by visualization with a Leica DM4000B microscope. Finally, the proportion of positive protein was calculated using ImageJ software.

### Western blot

Total protein was isolated using a total protein isolation kit (Solarbio, China) and its concentrations were estimated with the BCA method (Solarbio, China). Proteins (30 μg/per well) were applied for electrophoresis (5%∼10% SDS-PAGE) and then transferred onto polyvinylidene difluoride membranes (0.2 µM, Bio-Rad, USA). Subsequently, the PVDF membrane was blocked for 1.5 h at room temperature and then incubated with primary antibodies against iNOS (1:1000, ab178945), Arg1 (1:1000, CST93668), SIRT1 (1:1000, ab189494), acetyl-NF-κB p65 (Lys310) (1:1000, CST3045), phospho-NF-κB p65 (Ser536) (1:1000, CST3033), NF-κB-p65 (1:1000, CST8242), β-actin (1:2000, ab8226) overnight at 4 °C, and then incubated with secondary antibodies (1:3000, CST7074) for 1 h. Enhanced chemiluminescence (170-5070, Bio-Rad, USA) was applied for blots developing and the density was calculated by ImageJ system.

### Statistical analysis

Values were expressed as mean ± SD and were analysed by one-way ANOVA followed by the Bonferroni *post hoc* test by Graph Pad Prime 8.0 (La Jolla, USA). *P* value <0.05 was considered statistically significant.

## Results

### NMN improved cell viability and apoptosis status of LPS-treated MH-S cells

**Figure 1.**
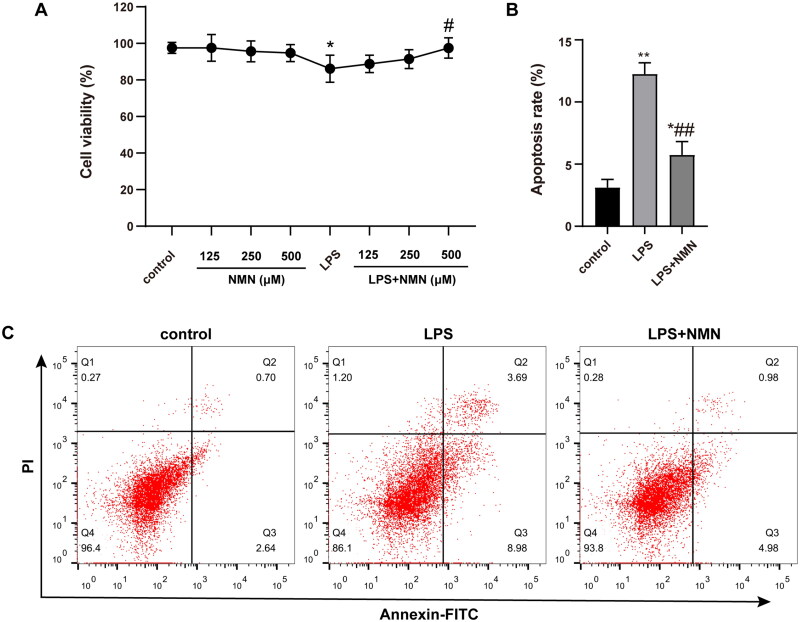
Effect of NMN on cell proliferation and apoptosis of LPS-stimulated MH-S lines. (A) Cell viability of differentially treated MH-S cells was determined by CCK8 kit. (B–C) Apoptosis of MH-S cells induced by different groups as indicated was measured by Annexin V-FITC/PI. Data are portrayed as mean ± SD and statistical analysis was performed by one-way ANOVA followed by the Bonferroni *post hoc* test. *p < 0.05, **p < 0.01 vs. controls, ^#^*p* < 0.05, ^##^*p* < 0.01 vs. LPS group.

To assess the potential cytotoxicity of NMN and its pretreatment on the LPS-treated MH-S cells, cellular viability was determined. The results suggested that the proliferative capacity of MH-S cells was unaffected by NMN at concentrations between 125 and 500 μM. Significantly decreased cell viability was observed after LPS stimulation, but when 500 μM NMN was incubated in the LPS group, there was a marked improvement in cell viability. In the settings, a dose of 500 μM NMN was used for the subsequent experiments *in vitro* (Figure 1(A)).

Apoptosis represents a critical feature of acute lung injury. LPS stimulation increased the apoptotic rate to 12.25%, but apoptosis was greatly alleviated by NMN treatment, leading to a significantly decreased apoptotic rate to 5.74% (Figure 1(B,C)).

### NMN regulated macrophage polarization from M1 to M2 phenotype in LPS-treated MH-S cells

**Figure 2.**
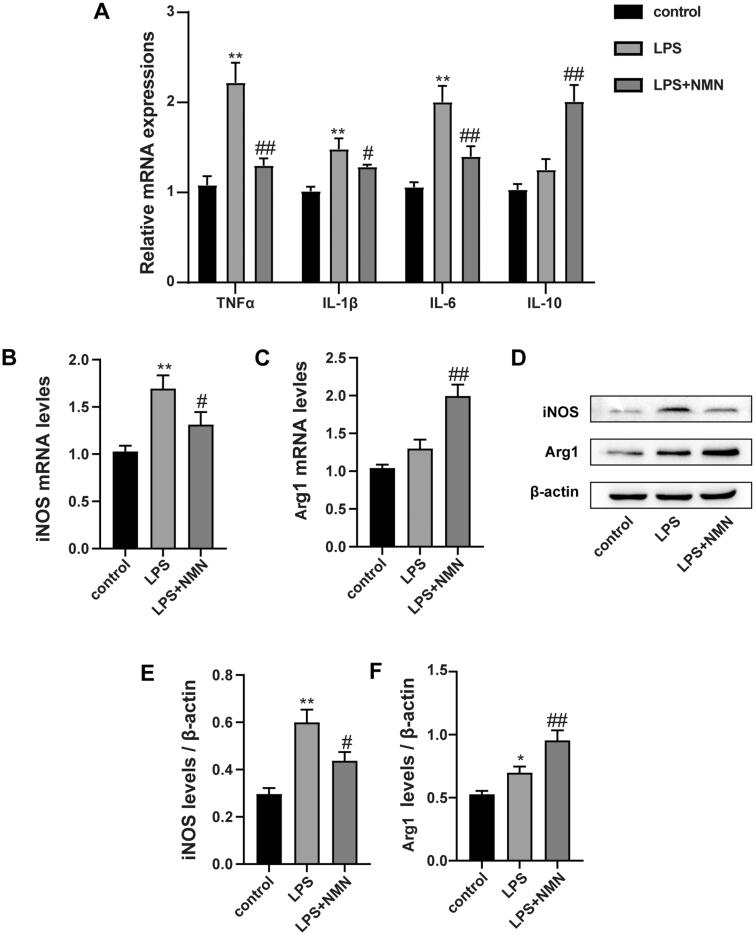
NMN promotes M2-type macrophage polarization in LPS-stimulated MH-S cells. (A) Relative mRNA expressions of IL-1β, TNF-α, IL-6 and IL-10 were detected. (B) Representative mRNA levels of iNOS. (C) Representative mRNA levels of Arg1. (D–F) Representative blots and relative expressions of iNOS and Arg1 in MH-S cells. Data are presented as mean ± SD and analysed by one-way ANOVA corrected with Bonferroni test. *p < 0.05 and **p < 0.01 relative to controls, ^#^*p* < 0.05 and ^##^*p* < 0.01 relative to LPS group, respectively.

Mounting studies point to a critical role of macrophage polarization in the development of sepsis and sepsis-induced organ injury. We thus measured the expression of inflammatory mediators and macrophage polarization related biomarkers. RT-PCR and immunoblotting analysis showed that administration of NMN prevented the LPS-induced M1 phenotype marker expression (pro-inflammatory cytokine IL-1β, TNF-α, IL-6, and iNOS) (Figure 2(A,B,D, E)), but elevated the M2 phenotype related markers (anti-inflammatory mediators IL-10 and Arg1) (Figure 2(A,C,D,F)). These results suggest that NMN incubation could polarize macrophages to an M2-like phenotype.

### NMN alleviated endotoxin-induced acute lung injury in mice

**Figure 3.**
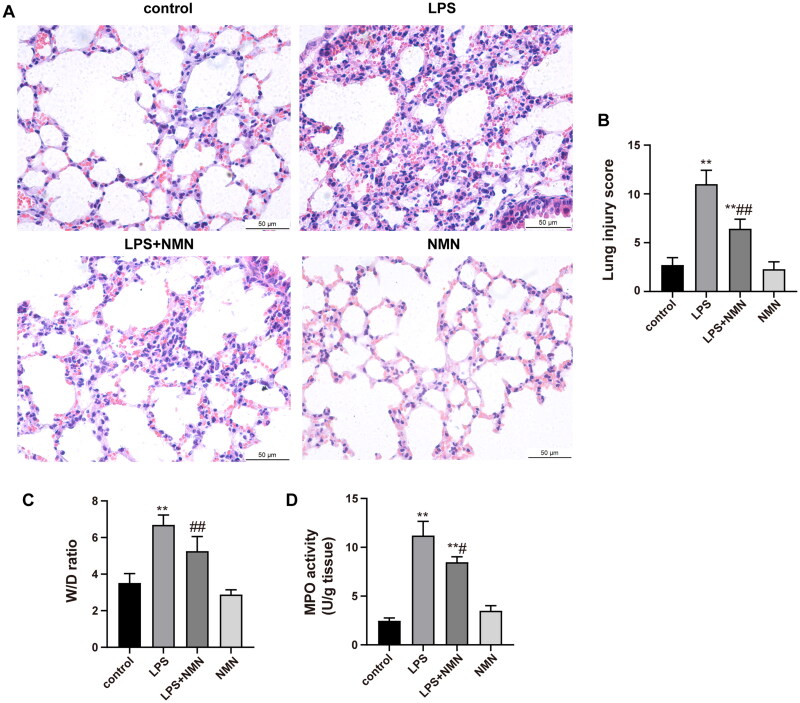
NMN attenuates lung injury induced by LPS. (A) The image of histopathologic changes of lung sections with H&E staining (×400). (B) Semiquantitative assessment of ALI using lung injury scores. The grading scale of 0 = minimal damage, 1+ =mild damage (25%), 2+ =moderate damage (50%), 3+ =severe damage (75%), and 4+ =maximal damage (almost 100%). (C) The W/D ratio. (D) MPO activity. Data are presented as mean ± SD and analysed by one-way ANOVA followed with Bonferroni test. *p < 0.05, **p < 0.01 vs. control and ^#^*p* < 0.05, ^##^*p* < 0.01 vs. LPS group.

To observe the effect of NMN on endotoxin-induced ALI, NMN (500 mg/kg, i.p.) was administered 1 h prior to LPS injection. Photomicrographs in the LPS group revealed explicit pathological alterations, including thickened alveolar wall, inflammatory cell infiltration, septa swelling, and erythrocyte exudation, which were dramatically ameliorated in the LPS + NMN group (Figure 3(A)). Consistently, semiquantitative lung injury scores showed that NMN pretreatment significantly decreased the scores relative to mice subjected to LPS (Figure 3(B)). In addition, the W/D ratio and MPO activity were also detected as indicators of lung oedema and neutrophil accumulation, respectively. Septic mice showed a noticeably higher W/D ratio and MPO activity relative to controls, while NMN administration suppressed the degree of pulmonary oedema and neutrophil recruitment (Figure 3(C,D)).

### NMN increased NAD^+^ and preserved bioenergetics

**Figure 4.**
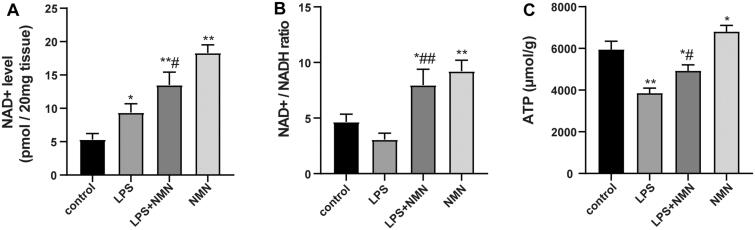
The impact of NMN on NAD^+^ and ATP levels in lung tissues. (A) The levels of NAD^+^ were determined with an NAD/NADH Assay Kit. (B) NAD^+^/NADH ratio in lung tissues. (**C**) ATP content was also detected in this part of the study. Data are expressed as mean ± SD using one-way ANOVA followed with Bonferroni test. *p < 0.05, **p < 0.01 vs. control and ^#^*p* < 0.05, ^##^*p* < 0.01 vs. LPS group.

Sepsis results in considerably fluctuating pyridine and adenine nucleotide pools, even depleted ATP reserves, which negatively affect the function of vital organs (Wei et al. 2021). Thus, we evaluated whether exogenous NMN could maintain NAD^+^ and ATP levels in lung tissues during endotoxin-induced ALI. Compared with controls, NMN significantly elevated NAD^+^, NAD^+^/NADH ratio, and ATP levels (Figure 4(A–C)). LPS caused a defensive increase in NAD^+^ levels but decreased NAD^+^/NADH ratio and ATP levels (Figure 4(A–C)). Notably, pretreatment with NMN markedly upregulated NAD^+^, NAD^+^/NADH ratio, and ATP levels, indicating that NMN increased NAD^+^ levels and restored ATP levels following LPS-induced ALI (Figure 4(A–C)).

### NMN polarized macrophages toward M2 anti-inflammatory profile in LPS-induced ALI

**Figure 5.**
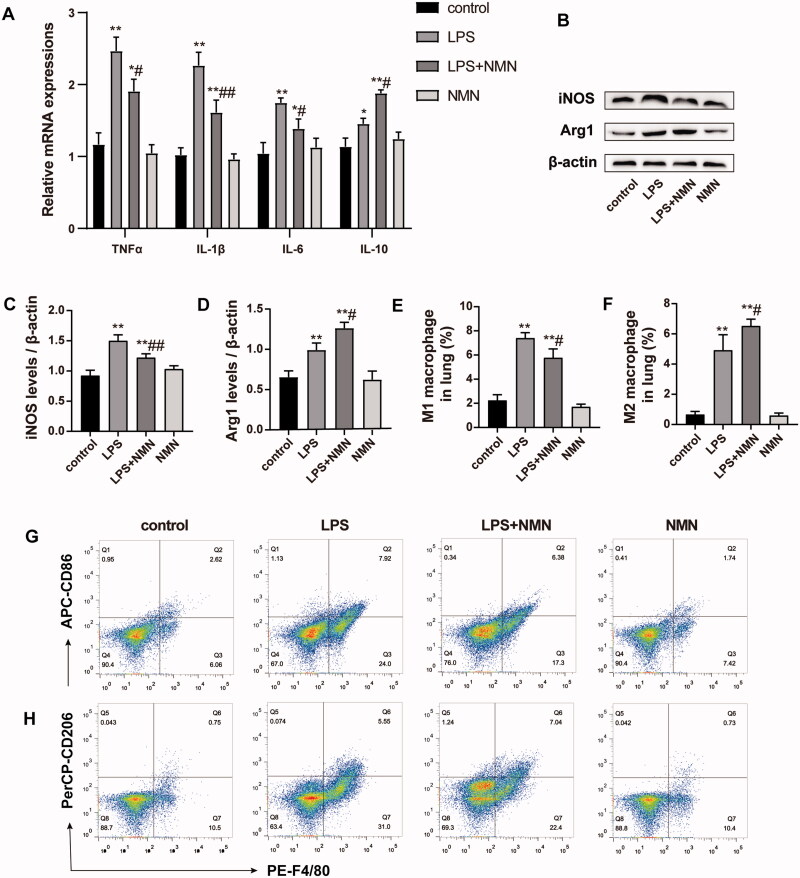
Effect of NMN on macrophage differentiation during LPS-induced ALI. (A) Relative mRNA levels of inflammatory cytokines IL-1β, TNF-α, IL-6, and IL-10 were determined. (B–D) Western blots of iNOS and Arg1 and quantitation. (E–G) The levels of CD86 (for M1 macrophages) and CD206 (for M2 macrophages) were evaluated by flow cytometric analysis. Values are expressed as mean ± SD and analysed by one-way ANOVA corrected with Bonferroni coefficient. *p < 0.05, **p < 0.01 vs. control group and ^#^*p* < 0.05, ^##^*p* < 0.01 vs. LPS group, respectively.

Based on the above observations, we next evaluated the impact of NMN on macrophage polarization and found that the levels of M1 macrophage-specific genes (IL-1β, TNF-α, and IL-6) were downregulated and the M2 macrophage-specific genes (IL-10) were upregulated in septic mice treated with NMN (Figure 5(A)). Apart from inflammatory mediators, NMN suppressed the increase in the expression of M1-related biomarker iNOS induced by LPS but promoted the expression of the M2-related biomarker Arg1 (Figure 5(B–D)).

Pulmonary macrophages were labelled as CD11b and F4/80 double-positive cells, in which CD86 and CD206 served as markers of M1 and M2 macrophages, respectively. Flow cytometric analysis intuitively indicated that NMN administration induced a highly pronounced anti-inflammatory M2 phenotype (CD206^+^ F4/80^+^) when mice were subjected to LPS while inhibiting the pro-inflammatory M1 phenotype (CD86^+^ F4/80^+^) (Figure 5(E–G)).

### NMN activated the SIRT1/NF-κB pathway in septic lung tissue

**Figure 6.**
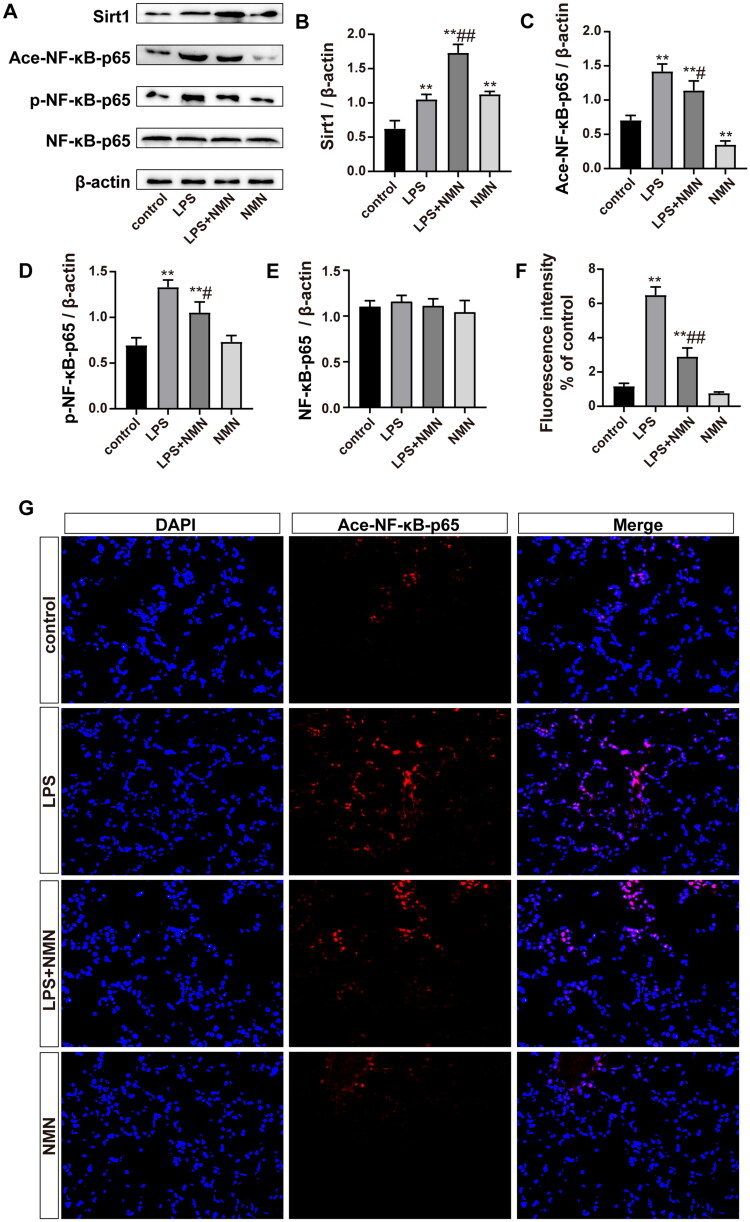
NMN activated the SIRT1/NF-κB pathway in sepsis-related ALI. (A–E) Representative bands and quantification of SIRT1, acetylated, phosphorylated NF-κB-p65, and NF-κB-p65. (F–G) Levels of acetylated NF-κB-p65 were shown by immunofluorescence (scale bar = 50 μm). Data are presented as means ± SD and analysed by one-way ANOVA followed with Bonferroni coefficient. **p < 0.01 compared with control; ^#^*p* < 0.05, ^##^*p* < 0.01 compared with LPS group.

To observe whether NMN alleviated ALI through the SIRT1/NF-κB pathway. First, immunoblotting showed that SIRT1 expression was significantly increased by LPS stimulation, which was consistent with the change in NAD^+^ levels (Figure 6(A,B)). Exogenous NMN upregulated SIRT1 protein levels with or without LPS treatment. As expected, immunoblotting and immunofluorescence showed that acetylated NF-κB-p65 was upregulated in septic mice, while NMN repressed acetylation (Figure 6(A,C,F,G)). Moreover, we also assessed the phosphorylation of NF-κB-p65, a critical proinflammatory pathway protein in alveolar macrophages and a sign of NF-κB activation. The results showed that NMN markedly suppressed the phosphorylation of NF-κB-p65 (Figure 6(A,D)). These findings strongly suggested that NMN acted *via* the SIRT1/NF-κB pathway to mitigate septic lung injury.

### The SIRT1/NF-κB signalling pathway was involved in NMN-mediated M2 macrophage polarization *in vivo*

**Figure 7.**
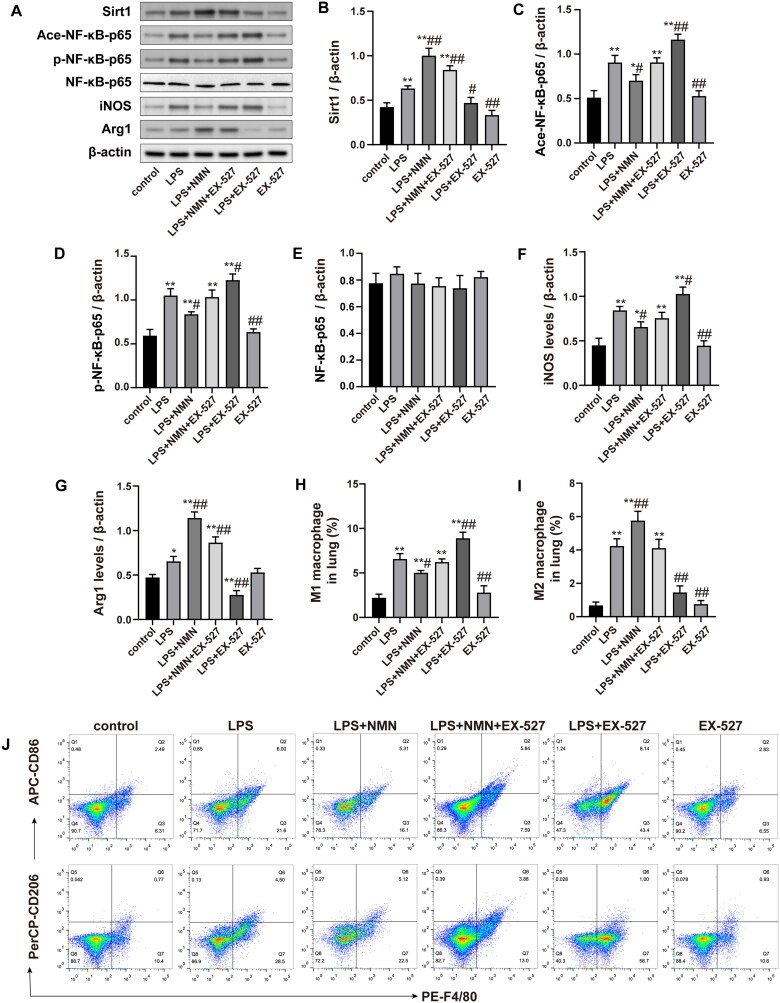
The SIRT1/NF-κB pathway was associated with the NMN-mediated M2 macrophage polarization. (A–G) Representative bands and quantification of SIRT1, acetylated, phosphorylated NF-κB-p65, NF-κB-p65, iNOS, and Arg1. (H–J) The levels of CD86 (for M1 macrophages) and CD206 (for M2 macrophages) were evaluated by flow cytometric analysis. Data are presented as means ± SD and analysed by one-way ANOVA followed with Bonferroni coefficient. *p < 0.05, **p < 0.01 compared with control; ^#^*p* < 0.05, ^##^*p* < 0.01 compared with LPS group.

To further determine whether SIRT1/NF-κB signaling activation was associated with NMN-regulated macrophage polarization, we examined the effect of SIRT1 inhibitor EX-527 on markers of M1/2 phenotype. As shown in Figure 7, EX-527 treatment decreased the expression of SIRT1, and enhanced expression of acetylated and phosphorylated NF-κB-p65 in septic mice pretreated with NMN (Figure 7(A–D)). In addition, SIRT1 inhibitor EX-527 also significantly promoted the expression of M1 macrophage-associated markers (iNOS and CD86) while inhibiting M2 phenotype expression (Arg1 and CD206) (Figure 7(A,F–J)). These results indicated that inhibition of SIRT1/NF-κB signaling reversed NMN-mediated M2 macrophage polarization.

## Discussion

Although the pathogenesis and potential therapies of endotoxin-induced ALI have been extensively recognized, optimal strategies to improve the resolution of inflammation and outcomes of patients remain to be determined. The present study demonstrated that NMN, an NAD^+^ precursor, exerted a potent anti-inflammatory effect and bioenergetic preservation. Additionally, NMN efficiently inhibited M1 macrophage polarization but promoted M2 polarization, and NMN targeted and regulated SIRT1/NF-κB signalling pathways thereby inhibiting the activation of NF-κB signaling and reducing inflammatory cascade. We further confirmed the beneficial pharmacological intervention of NMN on macrophage phenotype switching toward M2 *via* the SIRT1/NF-κB pathway.

NAD^+^ is a vital cofactor for multiple metabolic reactions that tightly impact mitochondrial bioenergetic metabolism and capacity, and accumulated studies have focused on the properties of DNA repair, anti-aging, and anti-inflammation (Rajman et al. 2018; Covarrubias et al. 2021; Navas and Carnero 2021; He et al. 2022). Notably, it has been demonstrated that the NAD^+^-related metabolite NMN could decrease the susceptibility to inflammatory disease and reduce oxidative stress (Guan et al. 2017; Wan et al. 2021; Zhang et al. 2021). Thus, further investigation will be required to identify the efficacy and detailed mechanisms of NMN in sepsis-induced ALI. Here, we observed that NMN could reduce the apoptosis and production of pro-inflammatory factor in LPS-stimulated MH-S cells and ameliorates pathologic lung injury in a murine septic model. NAD^+^ fluctuations and ATP depletion contribute to hallmarks of and driving forces behind sepsis (Suntharalingam et al. 2015; Hopp et al. 2021). NMN administration significantly upregulated NAD^+^ and ATP contents for maintaining mitochondrial function and cellular homeostasis. Collectively, our results identified the pivotal role of NMN in attenuating LPS-induced lung injury.

Macrophage polarization plays a prominent role in sepsis (Gong et al. 2019). M1 macrophages are characterized by secreting pro-inflammatory cytokines (TNF-α, IL-1β, and IL-6) and expressing hallmarks iNOS and CD86, then surge and amplify the “cytokine storm”, leading to organ damage (Wu et al. 2021). The M2 phenotype can produce anti-inflammatory factors (IL-10) and is labelled by specific markers CD206 and Arg1, which can resolve inflammation, promote wound healing, and suppress immunity (Wang et al. 2013). In this study, we found that the levels of pro-inflammatory cytokines and M1 markers robustly increased after LPS treatment. Conversely, NMN administration effectively inhibited the expression of M1-related markers and upregulated M2-related markers in response to LPS *in vivo* and *in vitro*.

Compelling evidence indicates that SIRT1 is a crucial dominator in the pathologies of sepsis as SIRT1 confers lung protection by inhibiting inflammation, apoptosis, oxidative stress, and fibrosis through the deacetylation of transcription factors and coactivators (Raji-Amirhasani et al. 2021). As NF-κB is a critical regulator of immune function, SIRT1/NF-κB signaling represents one of the critical cytoprotective mechanisms against various pathophysiological processes such as ulcerative colitis, neurological disorders, liver fibrosis, and sepsis (Liu et al. 2020; Shi et al. 2021; Shin et al. 2021; Tang et al. 2021). Notably, an obvious upregulation of SIRT1 protein was found in the LPS group contradictory to other studies (Mohamed et al. 2021), the defensive action which may be related to the dose and timing of LPS stimulation. Importantly, acetylated NF-κB-p65 was significantly increased under LPS stimulation, also the phosphorylated form of p65 was elevated, suggesting that NF-κB signalling was completely activated. However, NMN pretreatment increased the expression of SIRT1 but reduced the acetylation and phosphorylation of NF-κB-p65, suggesting that SIRT1/NF-κB signalling pathway might be involved in the lung protection of NMN. Furthermore, we found that EX-527 (a SIRT1 inhibitor) suppressed M2 polarization and exaggerated M1 polarization, which reversed the effect of NMN on macrophage phenotype. Therefore, we further confirmed that NMN promotes M2-like polarization by activating the SIRT1/NF-κB signaling pathway.

The current study has certain limitations. A plethora of evidence has demonstrated that substrates for SIRT1 deacetylase activity which include but are not limit to NF-κB-p65, deacetylation of p53, PGC1α, and FoxO, which are equally important for the resolution of inflammation. Thus, the underlying mechanism by which NMN attenuates ALI remains unclear and requires a more comprehensive study.

## Conclusions

NMN can effectively ameliorate sepsis-induced ALI through modulating macrophage polarization *via* SIRT1/NF-κB signalling pathway, which may provide a novel therapeutic direction for treating acute lung injury.
